# Listening in Noise Remains a Significant Challenge for Cochlear Implant Users: Evidence from Early Deafened and Those with Progressive Hearing Loss Compared to Peers with Normal Hearing

**DOI:** 10.3390/jcm9051381

**Published:** 2020-05-08

**Authors:** Yael Zaltz, Yossi Bugannim, Doreen Zechoval, Liat Kishon-Rabin, Ronen Perez

**Affiliations:** 1 The Department of Communication Disorders, Steyer School of Health Professions, Sackler Faculty of Medicine, Tel Aviv University, Tel Aviv-Yafo 6997801, Israel; yossi.bugannim@gmail.com (Y.B.); doreen.zechoval@gmail.com (D.Z.); lrabin@tauex.tau.ac.il (L.K.-R.); 2 Department of Otolaryngology and Head and Neck Surgery, Shaare Zedek Medical Center Affiliated to The Hebrew University Medical School, Jerusalem 9190501, Israel; perezro@inter.net.il

**Keywords:** Cochlear implant, hearing impairment, speech-in-noise, speech recognition, prelingually deafened, postlingually deafened, congenital hearing loss, progressive hearing loss, top-down processing, bottom-up processing

## Abstract

Cochlear implants (CIs) are the state-of-the-art therapy for individuals with severe to profound hearing loss, providing them with good functional hearing. Nevertheless, speech understanding in background noise remains a significant challenge. The purposes of this study were to: (1) conduct a novel within-study comparison of speech-in-noise performance across ages in different populations of CI and normal hearing (NH) listeners using an adaptive sentence-in-noise test, and (2) examine the relative contribution of sensory information and cognitive–linguistic factors to performance. Forty CI users (mean age 20 years) were divided into “early-implanted” <4 years (*n* = 16) and “late-implanted” >6 years (*n* = 11), all prelingually deafened, and “progressively deafened” (*n* = 13). The control group comprised 136 NH subjects (80 children, 56 adults). Testing included the Hebrew Matrix test, word recognition in quiet, and linguistic and cognitive tests. Results show poorer performance in noise for CI users across populations and ages compared to NH peers, and age at implantation and word recognition in quiet were found to be contributing factors. For those recognizing 50% or more of the words in quiet (*n* = 27), non-verbal intelligence and receptive vocabulary explained 63% of the variance in noise. This information helps delineate the relative contribution of top-down and bottom-up skills for speech recognition in noise and can help set expectations in CI counseling.

## 1. Introduction

Cochlear implants (CIs) are currently the gold-standard therapy of choice for individuals with severe to profound hearing loss who do not benefit from hearing aids. The CI device alters the auditory signal and transmits it via electrical pulses to the auditory nerve bypassing the inner ear [1,2]. The development of CIs revolutionized the therapy of hearing impairment, allowing adults with acquired hearing loss to successfully engage with their environment and enabling children born deaf to develop spoken language. While hearing-impaired cochlear implant users can achieve excellent performance in quiet, speech recognition in background noise continues to be a significant challenge for them, especially under conditions in which the target stimuli are not spatially separated from the noise [3,4,5,6,7,8,9,10]. This leads to significant communication problems in many real-life listening situations [11], and can negatively affect linguistic and cognitive development [6,12].

The difficulties that CI users experience in noise are mainly the result of the poor resolving capabilities of the CI device, leading to a degraded signal (poor “bottom-up” processing). In normal hearing (NH) listeners, the intact cochlea resolves the incoming signal to spectro-temporal cues that are perceived as pitch (F0), timing (speech onsets, offsets, and transitions between phonemes), and timbre (harmonics) [13,14], all of which contribute to the separation of speech from the noise. In contrast, the CI device, which presumably mimics the peripheral analysis of the incoming signal, is limited in the spectral information that it conveys as a result of the relatively small number of spectral channels [15] and its inability to resolve the temporal fine structure of the speech signal [16]. This, together with the relatively wide spread of electrically evoked neuronal excitation in the cochlea [17], produces vague representations of the spectro-temporal information required for phonemic perception [16,17].

It has been widely established that listeners cope with impoverished signals by resorting to linguistic and cognitive resources that assist them in the recognition of the message at the brain level (“top-down” processing) [18,19]. Phonemic and syntactic knowledge, for example, was found to assist in segregating the speech stream into syllables and words, whereas semantic knowledge allows the listener to make inferences about the content of the sentence and to constrain the possible responses [20,21]. At the same time, working memory is engaged [13] to store the speech signal long enough to solve possible mismatches between the degraded auditory input and previously encoded and stored information from the mental lexicon in long-term memory [22]. Some CI users with prelingual deafness (i.e., hearing loss that started at birth or at early age, before the acquisition of language), however, were shown to have poorer linguistic and/or cognitive skills compared to NH subjects [6,12,23,24,25,26,27,28,29,30], presumably because their brain was deprived of auditory stimulation prior to implantation [5,6,15,23,31,32,33], thus limiting their ability to exploit top-down predictive coding strategies for perception in noise [34,35]. Finally, personal background factors, including the age of hearing loss, use of residual hearing, mode of communication, and age at implantation, were also found to be contributing factors to the performance of speech-in-noise [18,22,24,36,37,38,39,40,41,42]. Age at implantation, for example, has been found to be strongly associated with performance in individuals with prelingual deafness [5,37,43,44], supporting the notion that early implantation during the “critical” or “sensitive” period in development efficiently restores cortical connectivity, allowing functional cross-modal brain reorganization [45,46,47] that may be necessary for the recognition of speech in noise. 

The majority of such studies have tested speech perception in noise in CI users who are postlingually deaf (i.e., individuals with acquired hearing loss who had normal acoustic hearing during their cognitive and language development) [10,40,48,49,50,51], with fewer studies assessing speech-in-noise recognition in prelingually deafened CI users (for review of studies see Appendix A Table A1 [3,4,5,6,8,32,37,52,53,54,55,56,57,58,59,60,61,62,63,64]. In general, participants with prelingual deafness have shown poor speech-in-noise performance, with a disadvantage of up to 20 dB in speech reception thresholds in noise (SRTn; SRTn is the signal-to-noise ratio (SNR) at which 50% of the words in noise are repeated correctly) compared to NH individuals [8]. Bugannim et al. [3], for example, reported that the average SRTn of young adults with CI was 9.45 dB SNR worse than that of their NH peers. Ching et al. [5] showed that the average SRTn of 5-year-old CI children was considerably poorer than that of NH children on a similar task (4.0–6.9 versus −1.2 dB SNR, respectively) [65]. Many studies, however, have tested speech-in-noise using a fixed, pre-determined SNR (e.g., [37,56]), making it difficult to compare performance between listeners with different listening abilities due to limitations such as having a minimal performance (“floor” effect) or reaching an optimal score (“ceiling” effect when reaching 100% correct) for poor and good performers, respectively, at a given SNR. Studies have also varied in the test stimuli. Some have used monosyllabic consonant–vowel–consonant (CVC) or spondee words [53,56], which can be either difficult or easy to perceive, depending on the CI user, and are considered less “ecological” as they do not reflect real-life communication. A few studies have examined sentence recognition in noise using an adaptive SNR [3,5,60], allowing for the documentation of a range of performance. However, these studies were limited to a single age group (children: 5, 60; adults: 3), and only two studies compared CI to NH [3,60]. Thus, the differences in the methodology used in the reported studies limit the ability to draw conclusions across different age groups or in comparison to NH peers, especially in children. Such comparisons are important for understanding the limitations of the CI device, setting realistic expectations, understanding the variability in CI performance, and developing future devices.

The purpose of the present study was twofold: (1) to conduct a within-study comparison of speech-in-noise performance in early-implanted and late-implanted CI users, including children, adolescents, and adults with prelingual and progressive hearing loss who had diverse listening abilities, and to compare their performance to NH peers; and (2) to examine the contribution of linguistic, cognitive, and background factors to performance by using a variety of tests that reflect processing in each of these domains. In order to obtain comparable speech-in-noise results across listening abilities and with respect to other reported studies, we chose to assess speech-in-noise perception using the Matrix sentence-in-noise test (Hebrew version [3]; for a review, see Kollmeier et al. [66]). This test comprises sentences with a fixed grammatical structure that are syntactically identical but semantically unpredictable, making the results less dependent on linguistic knowledge. In the Hebrew version that was used in the present study, the words are suitable for 5-year-old participants. Moreover, the sentences are presented with an adaptively changing SNR (a fixed noise level and adaptively changing speech level), making the test compatible with different levels of performance with high test–retest reproducibility [3,67].

## 2. Materials and Methods

### 2.1. Participants

Forty CI users (age range: 9.1–32.3 years, M = 19.9 ± 7.1) participated in the present study. There were no additional risk factors for developmental delays other than hearing loss in this group. These participants were divided into three subgroups: (1) “early-implanted” (*n* = 16), subjects who were implanted under the age of 4 years (participants CI1–CI16 in Table 1; M = 17.8 ± 5.6), of whom seven participants were implanted before the age of two years; (2) “late-implanted” (a similar definition of early and late implanted was used in Zaltz et al. [68]; *n* = 11), subjects who were implanted after the age of six years (participants CI17–CI27 in Table 1; M = 27.2 ± 3.8). These two subgroups included only prelingually deafened individuals. The third group comprised subjects defined as (3) “progressive” (*n* = 13), who had a progressive hearing loss (participants CI28–CI40 in Table 1; M = 16.3 ± 6.5), with two participants implanted before the age of four years. Note that the participants in the late-implanted group were found to be statistically older than those in the other two groups (F (2,39) = 13.262, *p* < 0.001). Half of the CI participants (*n* = 20) were bilateral CI users who were sequentially implanted. Thirty-two of the participants used Cochlear devices (19 had their first implant from the new generation of devices, and 13 had their first implant from the old generation), five participants used Med-El devices, and three used Advanced Bionics devices (see Table 1 for details of device). All had used hearing aids prior to implantation and used spoken language as their primary mode of communication. Detailed demographic data for this group are shown in Table 1. In addition, 136 NH participants (age range: 7.9–29.9 years; M = 17.2 ± 7.4) served as a control group, including 80 children and adolescents (M = 11.3 ± 2.5) and 56 young adults (M = 25.6 ± 2.0).

All the participants were native Hebrew speakers. All adults and parents of the children had at least 12 years of education. Informed consent was obtained from all the adults and from the parents of the children who participated in the study. The study was approved by the Institutional Review Board of Ethics at Tel Aviv University and by the human experimentation ethics committee (Helsinki Committee, Number: 0258/17) of Shaare Zedek Medical Center.

### 2.2. Hebrew Version of the Matrix Sentence-In-Noise Test

Sentence recognition in noise was estimated using the Hebrew version of the Matrix sentence-in-noise test [3]. This test consists of sentences that have the same grammatical structure (in Hebrew: Name–verb–number–noun–adjective) and employs a base list of 50 words (appropriate for 5-year-old children), with 10 words in each grammatical category. Theoretically, the 50 words, recorded by a native Hebrew-speaking female talker, can make up to 100,000 different sentences. The noise was a steady-state speech-shaped noise, which was generated by superimposing all synthesized sentences [66]. Note that the optimization procedure was done at Oldenberg University to ensure that the test was similar in all languages. The noise was presented at a fixed level of 65 dB sound pressure level (SPL), and an adaptive procedure was used to estimate SRTn [69]. Specifically, at first presentation, the sentence was presented at SNR = 0 dB. The listeners were asked to orally repeat everything they heard, as accurately as possible, and were encouraged to guess in cases of uncertainty. There was no time limit for response. On the basis of the listener’s answer, the tester indicated the words that were correctly recognized on the computer, and the level of the next sentence was varied; correct word recognition of 1, 2, 3, 4, or 5 words resulted in the presentation of the next sentence at the following SNR levels: +4.5, +1.5, −1.5, −4.5, and −7.5 dB, respectively. The step size decreased exponentially after each reversal of the presentation level. The final SRTn was estimated using a maximum-likelihood procedure [69] based on 20 different sentences.

### 2.3. Word Recognition in Quiet

Word recognition in quiet was assessed using the Hebrew version of the Arthur Boothroyd (AB) [70] word recognition test (HAB) [71]. This test includes open-set monosyllabic, consonant–vowel–consonant isophonemic word lists. There are 10 words in each list, where each consonant appears once, and each vowel appears twice. The participants were required to orally repeat two-word lists that were presented at 65 dB SPL in quiet. There was no time limit for response. Performance was calculated as the percent of correctly identified words.

### 2.4. Language Assessment

Receptive vocabulary was estimated using a picture test. The examiner said words in Hebrew, and the participant was required to point to one of four pictures that matched each spoken word. The items included verbs, adjectives, and nouns, and were arranged in several sets with increasing difficulty based on the words’ prevalence in Hebrew. There was no time limit for response. The start set differed between participants on the basis of age, with older participants starting with a more difficult set. However, if the participant provided two or more incorrect responses in the start set, an easier set was presented. Testing ended when the participant gave more than seven incorrect responses within a single set. The vocabulary score was calculated as the percent of correctly identified words, assuming the correct identification of words in sets that were easier than the start set. Phonemic fluency was assessed by asking the participants to provide as many words as possible within 60 s for each of three letters in Hebrew: bet (/b/), gimel (/g/), and shin (/š/) [72]. Semantic fluency was assessed by asking the participants to provide as many words as possible within 60 s in each of three categories: animals, fruits and vegetables, and vehicles, regardless of the initial letter [72]. The phonemic/semantic fluency score was calculated as the mean number of words generated in one minute for the three letters/categories, respectively.

### 2.5. Cognitive Assessment

Non-verbal reasoning was assessed using the Raven’s Standard progressive Matrix test [73]. The children and adults saw 24 or 60 visual patterns with a missing piece, respectively, and were required to select one of six or eight patterns in order to correctly complete the visual display. The adults and older children used the computer mouse, whereas younger children provided an oral response. The Raven score was based on the relative percentage of correctly completed patterns.

Auditory working memory was assessed using the backward digit span subtest of the Wechsler Intelligence Scale [74]. The participants heard sequences of numbers (e.g., 2, 6, 4, and 3) and were asked to orally repeat them in the reverse order. The passing criterion to proceed to the next longer sequence was one successful repetition of a sequence of a specific length. The score represented the number of correctly repeated sequences.

Visual attention and perceptual speed of processing were assessed using the Trail Making test (TMT) part A [75]. In this test, the participants were instructed to manually connect, by drawing a line as quickly as possible, a set of 24 consecutive numbers in sequential order while still maintaining accuracy. If a participant made an error, the tester corrected the response before moving on to the next dot. The TMT score represented the time taken for the participant to complete the test accurately (in seconds).

### 2.6. Apparatus

All testing took place in a soundproof room. Stimuli were delivered using a laptop personal computer through a loudspeaker that was located 1 m in front of the participant. Bilateral CI users were tested wearing both CIs, whereas bimodal listeners were tested only with their CI device (hearing aid turned off). NH listeners were tested monaurally via Sennheiser HDA-200 headphones.

### 2.7. Study Design

All the participants took part in a single testing session. Each participant listened to four Matrix lists, with 20 sentences in each list. The first two lists were used to familiarize the subject with the task [76], and the last two were taken for the SRTn. In addition to the Matrix testing, word recognition in quiet and linguistic and cognitive abilities were assessed in a semi-randomized order. Note that not all the participants were tested in all the additional measures: HAB was tested in 39 CI users, receptive vocabulary was tested in 23 CI users, and fluency tasks were tested in 24 CI users. Raven’s standard progressive Matrix test was tested in 34 CI users and 71 NH listeners, TMT was tested in 37 CI users and 135 NH listeners, and backward digit-span was tested in 38 CI users and 114 NH listeners.

### 2.8. Data Analysis

Statistical analyses were conducted using the Statistical Package for the Social Sciences (SPSS) software version 20. Significance was set at 0.05. All post-hoc analyses were conducted using Bonferroni corrections for multiple comparisons. Sentence recognition in noise was assessed as the mean of measurements 3 and 4 of the Matrix test; this method is based on previous studies that used a similar measure [3,66] and on preliminary analysis that showed no significant effect of measurement (3, 4) on performance (*p* > 0.05).

## 3. Results

### 3.1. Sentence Recognition in Noise

**Figure 1:**
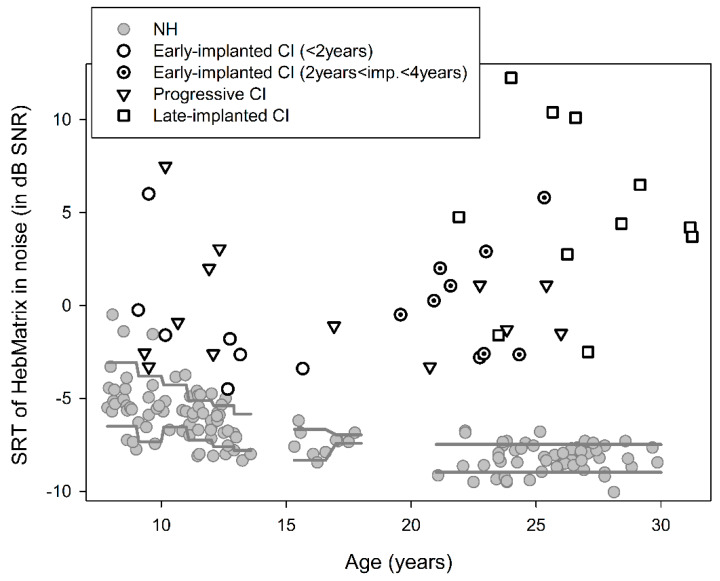
Individual speech reception thresholds in noise (SRTn) of the Hebrew Matrix sentence-in-noise test (mean signal-to-noise ratio (SNR) in measurements 3, 4) for early-implanted cochlear implants (CI) users (*n* = 16, seven implanted before two years of age), progressive CI users (*n* = 13), late-implanted CI users (*n* = 11), and normal hearing (NH) controls (*n* = 136). Mean performance of the NH ±1 standard deviation is shown between the gray lines.

The individual results in the Matrix test (mean measurements 3, 4) for the three subgroups of CI users (early-implanted, progressive, late-implanted) and the NH controls are shown in Figure 1. A large between-subject variability can be detected in the CI group, with SRTn values ranging from −4.5 to +12.25 (a range of approximately 17 dB SNR), compared to values from −10.05 to −0.5 (a range of 9.5 dB SNR) in the NH group. In addition, a clear disadvantage was evident for most CI users, and especially for the late-implanted ones compared to the NH listeners. That is, most CI users needed a larger SNR in order to reach SRTn, with a few early-implanted CI children showing similar performance to that of the poorest performing NH children.

**Figure 2:**
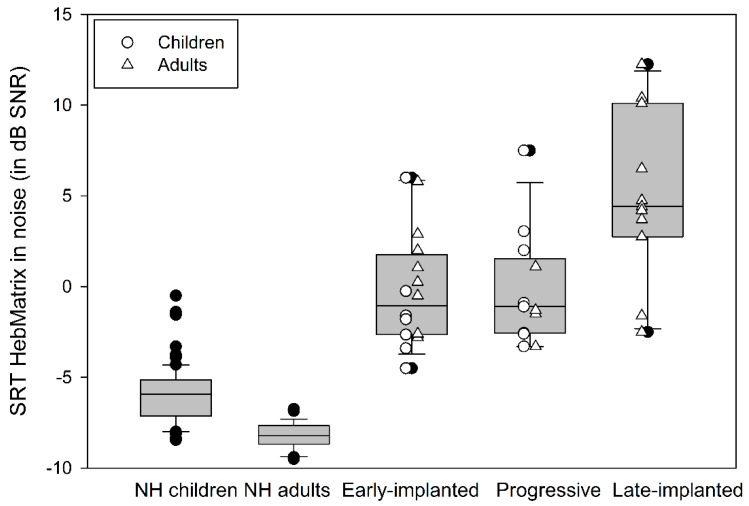
Box-and-whisker plot of the SRTn of the Hebrew Matrix sentence-in-noise test (mean SNR in measurements 3, 4) for early-implanted CI users (*n* = 16), progressive CI users (*n* = 13), late-implanted CI users (*n* = 11), and NH controls (children: *n* = 80, adults: 56). Also shown are the individual results of the children (empty circles) and adult (empty triangles) CI users. Note that within the “early-implanted” CI group, the children were implanted before two years of age.

In order to compare performance in noise across the three subgroups of CI users, univariate analysis was conducted with Subgroup (early-implanted, late-implanted, progressive) as a fixed factor and Age and Generation of the first implanted CI device as covariates. Results showed a significant main effect of Subgroup (F(2,35) = 4.678, *p* = 0.016, ƞ^2^ = 0.211), with no significant effects of Age (F(1,35) = 0.130, *p* = 0.721) or Generation of the CI device (F(1,35) = 1.007, *p* = 0.323). Post-hoc analysis showed significantly worse thresholds for the late-implanted subgroup (M = 5 ± 4.66) compared to the early-implanted (M = −0.3 ± 3.15; *p* = 0.005) and the progressive (M = −0.14 ± 3.03; *p* = 0.008) subgroups. No significant difference in thresholds was found between the latter two subgroups (*p* > 0.99). Figure 2 shows box-and-whisker plots of the SRTn of the early-implanted, late-implanted, and progressive CI users. For comparison purposes, the distributions of the SRTn for the NH children and adults are also shown.

In order to compare performance in noise between the early-implanted and progressive (E&P) CI users and the NH listeners, univariate analysis was conducted with Group (E&P, NH) as a fixed factor and Age as a covariate. Results show significant main effects of Group (F(1,162) = 363.705, *p* < 0.001, ƞ^2^ = 0.692), with worse thresholds for the E&P group (M = −0.23 ± 3.05, −6.90 ± 1.69, for the E&P and the NH, respectively) and Age (F(1,162) = 59.959, *p* < 0.001, ƞ^2^ = 0.270), with worse thresholds as age decreased.

### 3.2. Word Recognition in Quiet

**Figure 3:**
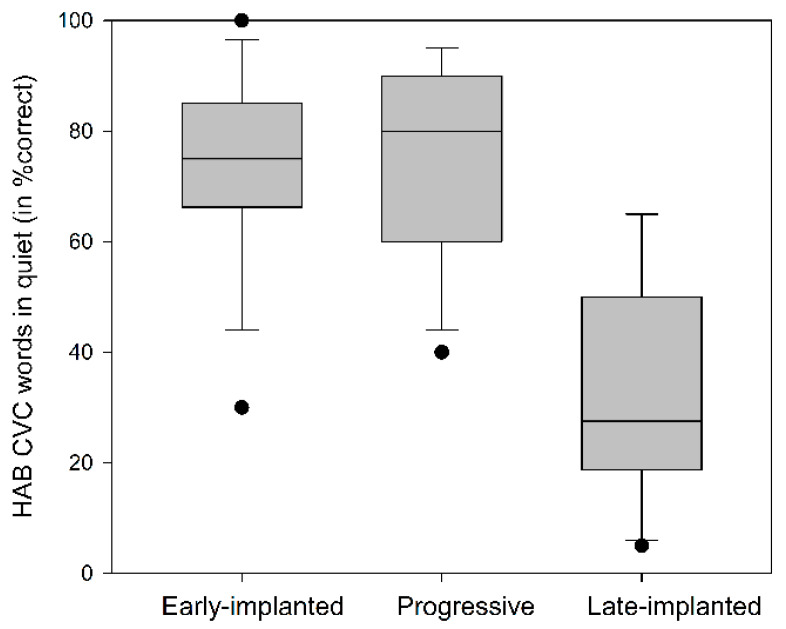
Box-and-whisker plot of the results in the Hebrew Arthur Boothroyd (AB) consonant–vowel–consonant (CVC) words in quiet (HAB) test for the early-implanted (*n* = 16), progressive (*n* = 13), and late-implanted (*n* = 10) CI users.

**Figure 4:**
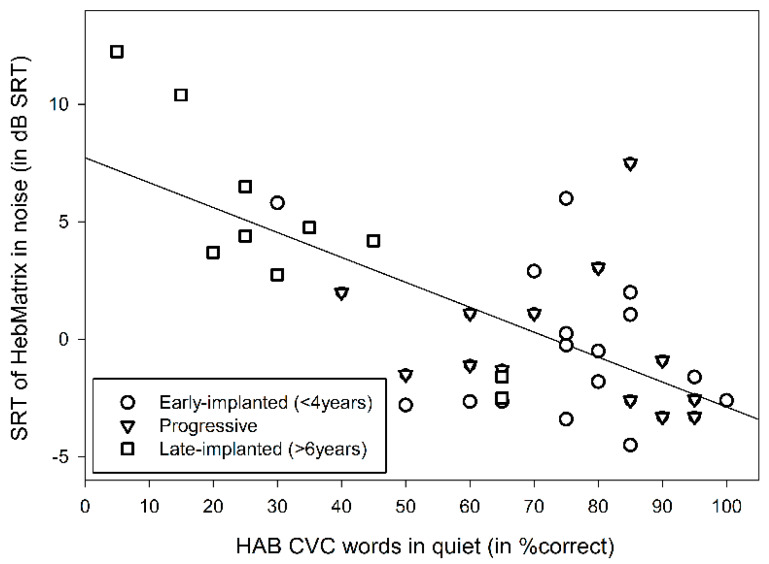
Individual results in the Hebrew CVC words in quiet (HAB) test versus SRTn of the Hebrew Matrix sentence-in-noise test (mean SNR in measurements 3, 4), for the early-implanted (*n* = 16), progressive (*n* = 13), and late-implanted (*n* = 10) CI users.

The univariate analysis conducted for the HAB results of the CI group with Age and Generation of the first implanted CI device as covariates revealed a significant effect of Subgroup (F(2,38) = 6.55, *p* = 0.004, ƞ^2^ = 0.272), with worse results for the late-implanted CI users (M = 33 ± 20.03) compared to the early-implanted (M = 74.06 ± 17.05; *p* = 0.003) and progressive (M = 74.23 ± 18.01; *p* = 0.012) subgroups, and no significant difference was found between the latter two subgroups (*p* > 0.99) (Figure 3). There was also a significant effect of Age (F(1,38) = 4.704, *p* = 0.037, ƞ^2^ = 0.122), with worse thresholds as age increased, with no significant effect of Generation of the CI device (F(1,38) = 0.113, *p* = 0.739). It can also be seen from Figure 3 that there was large variability in performance in each of the subgroups. Individual HAB data as a function of SRTn (Figure 4) shows that scores for word recognition in quiet of less than 50% and of more than 90% appear to be closely associated with the SRTn, whereas for scores between 50% and 90%, the SRTn results are variable. Pearson coefficient correlation revealed a significant association between the HAB and Matrix results (r = −0.669, *p* < 0.001), with word recognition in quiet explaining 44.75% of the variance of sentence recognition in noise.

### 3.3. Linguistic and Cognitive Factors

Table 2 shows the average results of the linguistic and cognitive tests, which are presented separately for each subgroup of CI users. It can be seen that, in general, there was a trend toward better performance in the phonemic fluency test for the late-implanted subgroup compared to the early-implanted and progressive subgroups. This trend was probably influenced by the older age of participants in the late-implanted subgroup, as phonemic fluency is expected to improve with age [72]. Multivariate analysis reveals no significant differences between the subgroups on any of the tests (*p* > 0.05).

As Figure 4 indicates that word recognition of more than 50% is less associated with SRTn, we further examined the contribution of the cognitive and linguistic factors for 29 participants who showed better than 50% correct word recognition in quiet (termed Q50 performers). This subgroup included 15/16 early-implanted, 2/11 late-implanted, and 12/13 progressively deafened CI participants. Pearson coefficient correlations conducted between the speech-in-noise Matrix results and the cognitive and linguistic factors revealed significant correlations between the SRTn and the Raven score (r = −0.548, *p* = 0.004), the receptive vocabulary score (r = −0.644, *p* = 0.003), and the phonemic fluency score (r = −0.522, *p* = 0.022). No significant correlations were found between the SRTn and the Wechsler backward digit-span test, the TMT, or the semantic fluency test (*p* > 0.05). A stepwise regression analysis was conducted on the factors that were found to correlate with the Matrix results, and the results show that the Raven score explained 38% of the variance, and the Raven plus receptive vocabulary score explained a total of 62.9% of the variance in the SRTn of the Q50 group (*p* < 0.05). No significant correlation was found between the Raven and the receptive vocabulary scores (*p* > 0.05), suggesting that both were independent predictors of performance in noise. A similar analysis that was conducted for the NH groups reveals no significant correlations between the Raven score and the SRTn for either the children or adults (*p* > 0.05).

**Figure 5:**
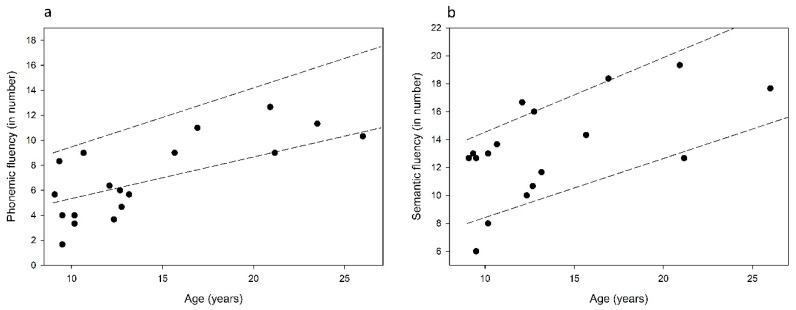
Individual scores in the (**a**) phonemic and (**b**) semantic fluency tests for the Q50 performers (CI users who scored ≥50% in the words-in-quiet test) compared to NH performance (shown between the broken lines: mean ± Standard deviation by age) from Kave and Knafo-Noam [72].

Independent sample *t*-tests were conducted to compare cognitive performance between the Q50 performers and the NH controls. These were conducted separately for the children (15 CI and 80 NH) and adults (14 CI and 56 NH). Results showed no significant difference between the groups in any of the cognitive measures that were tested (*p* > 0.05). As our NH controls were not tested on the linguistic tasks, performance in the fluency tests of Q50 performers was compared to that known for NH from the literature. Figure 5a,b shows the individual phonemic and semantic fluency scores of the Q50 performers compared to NH from Kave and Knafo-Noam [72]. Results showed that 42% (8/19) and 79% (15/19) of the Q50 performers performed within the range of NH performance (mean ± standard deviation) in the phonemic and semantic fluency tasks, respectively.

## 4. Discussion

The present study provides, for the first time, a comparative view of speech-in-noise performance across different populations of CI users and NH subjects of different ages using a sentence-in-noise test (Hebrew Matrix). The results of the study support the following findings. First, the CI users showed poorer sentence recognition in noise as compared to the NH listeners (a mean disadvantage of 5.29 dB for the children and adolescents and 10.4 dB for the adults in SNR). The best CI performers failed to achieve SRTn within the range (mean ± 1 standard deviation) of NH subjects of comparable ages. Second, there was large variability in the speech-in-noise performance of the CI users, whereby the congenitally deafened who were early-implanted and those with progressive hearing loss showed better word recognition in quiet and sentence recognition in noise compared to the congenitally deafened who were late-implanted. Third, word recognition in quiet predicted speech-in-noise performance, more so for the worst (<50% word in quiet) and best (>90% word in quiet) performers. Fourth, non-verbal intelligence and receptive vocabulary explained 63% of the variance in speech-in-noise results for better-performing CI users, i.e., those who achieved above 50%word recognition in quiet (*n* = 29).

The finding that the performance in noise of CI users across subgroups was inferior to that of their NH peers, requiring an SNR that was 5–10 dB higher in order to achieve 50% correct sentence recognition, is consistent with previous reports [3,4,6]. This disadvantage was found to be strongly related to two main factors: age at implantation and word recognition in quiet. Age at implantation, which reflects the period of hearing deprivation for prelingually deafened individuals, was found to be a significant predictor for speech-in-noise perception in the present study, with the late-implanted CI users requiring on average about 5 dB SNR to achieve 50% correct sentence recognition compared to about −0.3 dB and −0.14 dB for the early-implanted CI users and CI users with progressive hearing loss, respectively. Furthermore, within the early-implanted group, there was a tendency toward better performance for those implanted before 2-years-old compared to those implanted after 2-years-old, with the first requiring an SNR of −1.2 dB to achieve 50% correct sentence recognition compared to 0.38 dB for the latter (Figure 1). These findings are in accordance with a recent study that reported an average SRTn of −0.2 dB SNR with CVC words for 4–6-year-old children who were implanted before the age of two compared to 3.4 dB SNR for children at the same age who were implanted between two and five years of age [52]. Noteworthy is the fact that the participants who were implanted under 2-years-old in the present study were children or adolescents at the time of testing, whereas those implanted between 2- and 4-years-old were adults. In general, NH children are expected to show less mature speech-in-noise perception compared to NH adults because of less developed top-down capabilities [13], as found in the present study. However, in our early-implanted CI group, there was a reversed trend. That is, the children and adolescents slightly outperformed the adults, further emphasizing the positive effect of a shorter hearing deprivation period on hearing performance in noise [38,42,65].

Monosyllabic word recognition in quiet was a significant contributing factor to the SRTn of the CI users, mainly in the late-implanted group. Specifically, in this group, results were significantly poorer (an average of about 33% correct for CVC words in quiet) compared to the early-implanted and progressive groups (an average of about 74% for each group), which is in line with other studies [77,78,79]. These results support the notion that a prerequisite for good speech-in-noise perception is good recognition of words in quiet. The finding that the late-implanted individuals performed poorly on word recognition in quiet suggests that their CI device did not transmit the necessary spectro-temporal information of the incoming auditory signal [3] and/or they could not efficiently exploit the transmitted information. The latter may have resulted from the fact that auditory stimulation was introduced after the sensitive period of increased plasticity in the auditory pathway, which allowed for some restoration of the tonotopic organization in the cochlea without sufficiently improving synaptic functionality for efficient input-driven processing [46,47].

Another novelty of the present study stems from the finding that a wide range of performance of SRTn (−4.5 to +7.6 dB) was found for participants who reached 50% correct or more word recognition in quiet (the Q50 performers), most of whom were from the early-implanted and progressive subgroups. Furthermore, for this group of CI users, variability in performance in noise was not significantly related to their word recognition in quiet. This may suggest that after reaching 50% word recognition in quiet, presumably reflecting the minimal necessary exploitable information from the CI device for sentence recognition in noise, further improvement in recognition is dependent on linguistic and cognitive skills. Our findings show that for these Q50 performers, non-verbal intelligence and receptive vocabulary explained close to 63% of the variance in performance in noise. It is possible that for these individuals, superior cognitive functions and/or good language skills helped to overcome the reduced sensory input. Alternatively, it may suggest that the good spectro-temporal analysis provided by their CI device(s) led to good language and cognitive outcomes. Non-verbal IQ measures were previously suggested to reflect fluid intelligence, including mental mechanisms that need to be engaged when an individual is faced with a task that cannot be performed automatically [80]. In challenging listening conditions, these mechanisms may comprise selective auditory attention, shifting, inhibition, and working memory skills that may help to extract the relevant signal features from the competing background and store them in memory [18]. Top-down predicting coding based on linguistic knowledge may then enhance the encoding of the distorted signal elements, with linguistic cues filling the missing acoustic details [13] and improving speech perception [13,35]. Note that the average cognitive performance of the CI users in the present study was within the range of their NH peers, in accordance with some previous reports [24,76]. Nevertheless, none of the Q50 performers reached speech-in-noise performance within 1 standard deviation of the mean of NH participants of comparable ages. The positive association found between cognitive abilities and speech-in-noise performance in the Q50 performers may therefore reflect the high cognitive demands involved in the restoration of the degraded speech signal provided by the CI device.

### Limitations and Suggestions for Future Research

One limitation of the present study is the fact that the late-implanted participants included only adults, whereas the early-implanted and progressive groups included children, adolescents, and adults. The fact that the CI children outperformed the CI adults suggests that this was not a confounding factor. A second limitation is that CI devices differed between participants, with some using older speech processors, which may have contributed to the variability in the results. These two methodological factors were addressed statistically by holding age and generation of the CI as covariates in the statistical analysis of the CI group. The generation of the CI device was not found to be a significant factor. Nonetheless, future studies should attempt to compare CI groups that are age-matched at the time of testing and who use CI devices with advanced speech processors. Note also that the majority of our participants (32 of 40) use Cochlear devices; therefore, the data reported are limited to one CI manufacturer. Future studies should include CI devices of different manufacturers in order to test the contribution of the different strategies of speech processing on performance in noise. Finally, the present study tested the association between top-down processing and sentence recognition in noise by assessing a limited set of cognitive and linguistic abilities. It is possible that other cognitive functions, such as auditory attention, inhibition, and learning ability, contribute to the recognition of speech-in-noise.

## 5. Conclusions

The present study is the first of its kind to compare sentence-in-noise data from a wide range of cochlear implant users (*n* = 40; prelingual early-implanted and late-implanted and those with progressive hearing loss) to data from normal hearing individuals (*n* = 136), from children to young adults, using a sentence-in-noise test (Matrix test) that has been adapted to 17 languages [66] and will thus be helpful in setting expectations worldwide. Our data help to delineate the relative contribution of top-down and bottom-up factors for sentence recognition in noise in a diverse population of cochlear implant users. Sentence recognition in noise is associated with word recognition scores in quiet when the latter is less than 50%, whereas linguistic and cognitive factors are significant contributors (explaining 63% of the variance of sentence recognition in noise) when performance in quiet is better than 50% recognition. This suggests that speech recognition in noise depends on receiving critical usable information from the device, after which more central factors associate with performance. Future studies with larger samples will allow us to quantify the relative contribution of each of the top-down and bottom-up processes for predicting speech-in-noise and to possibly tailor habilitation protocols accordingly. The current findings continue to support the notion that early access to auditory stimulation is associated with better hearing skills, including speech perception in noise. Nonetheless, the best of the CI users within the early-implanted group failed to reach 1 standard deviation of the mean of NH subjects of comparable ages. This suggests that major advancements in the technology of the CI device and in novel habilitation protocols have yet to overcome the challenge of listening to speech-in-noise.

## Figures and Tables

**Table 1:** Demographic details for the cochlear implant (CI) users who participated in the present study. Participants CI1–CI27 were prelingually deafened individuals. Of these participants, CI1–CI16 were early-implanted (before the age of 4 years), and CI17–CI27 were late-implanted (after the age of 6 years). Participants CI28–CI40 were individuals with progressive deafness.

Subject ID	Gender	Etiology	Age at Identification (Years)	Age at Fitting HA (Years)	Age at Implantation (Years)	Age at Testing (Years)	Implant
CI1	F	Suspected neonatal jaundice	Birth	0.5	1.1 (L) 1.5 (R)	10.17	Cochlear C512 (R + L)
CI2	F	Genetic	Birth	0.25	1.11 (R) 4.11 (L)	15.67	Cochlear Freedom (R + L)
CI3	F	Genetic	Birth	0.25	1 (R) 2 (L)	12.67	Cochlear Freedom (R + L)
CI4	F	Genetic	Birth	0.33	1.5 (R) 2.4 (L)	9.50	Cochlear C512 (R + L)
CI5	M	Genetic	Birth	0.58	1 (R) 5 (L)	13.17	Cochlear Freedom (R) C512 (L)
CI6	M	Unknown	Birth	0.58	1 (R) 1.75 (L)	9.08	Cochlear C512 (R + L)
CI7	M	Suspected CMV	Birth	0.5	1.11 (L)	12.75	Cochlear C512 (L)
CI8	F	Unknown	0.25	0.25	2.8 15.7	20.9	Cochlear Freedom (R + L)
CI9	M	Genetic-connexin	0.67	0.83	2.5 (L)	24.3	Cochlear Freedom (L)
CI10	M	Genetic	1.5	2	3 (L)	21.2	Cochlear Freedom (L)
CI11	M	Genetic	1.5	1.5	3.7 (R) 15 (L)	21.6	Cochlear Espirit (R) Freedom (L)
CI12	F	Waardenburg syndrome	Birth	Unknown	3 (L)	25.3	Cochlear Espirit (L)
CI13	F	Genetic	0.5	0.5	2.5 (L)	19.6	Cochlear Espirit (L)
CI14	F	Waardenburg syndrome	Birth	0.25	2.5 (L) 16 (R)	22.8	Cochlear Freedom (R) Nucleus 5 (L)
CI15	M	Meningitis	0.58	0.58	2.3 (R) 14 (L)	22.9	Cochlear Nucleus 22 (R) Nucleus 24 (L)
CI16	M	Genetic	0.83	1	3 (L)	23	Cochlear Nucleus (L)
CI17	M	Genetic	Birth	1.67	9 (R) 19 (L)	29.2	Cochlear Sprint (R) Freedom (L)
CI18	M	Genetic	Birth	1	6 (R) 13 (L)	23.6	Cochlear Sprint (R) Freedom (L)
CI19	M	Unknown	Birth	0.5	6 (L)	21.9	Cochlear Nucleus
CI20	M	Unknown	Birth	1	12.7 (R)	28.4	Cochlear Nucleus
CI21	F	Unknown	Birth	1	8.3 (R)	24	Advanced Bionics Naida
CI22	M	Genetic-Connexin	Birth	1	29.1 (L)	31.2	Advanced Bionics Naida
CI23	M	Genetic-Connexin	Birth	0.25	15.3 (R) 25.7 (L)	27.1	MedEL Opus (R + L)
CI24	F	Unknown	Birth	1	6.2 (L)	26.3	Cochlear Nucleus
CI25	M	Unknown	Birth	1	9 (L) 24.5 (R)	25.7	Cochlear Nucleus (R + L)
CI26	M	Suspected hepatitis	Birth	0.67	21.9 (L)	26.6	Cochlear Nucleus (L)
CI27	M	Unknown	Birth	1.5	31.33 (L)	32.3	MedEl Opus (L)
CI28	F	Genetic-Connexin	Progressive	Unknown	8.9 (R)	9.33	Cochlear C512 (R)
CI29	F	Unknown	Progressive	4	7.2 (R) 8.9 (L)	10.17	Cochlear C512 (R + L)
CI30	M	Genetic	Progressive	2	3.6 (R) 3.11 (L)	9.5	Cochlear C512 (R + L)
CI31	F	Genetic	Progressive	3	6.9 (L)	10.67	MedEl Rondo (L)
CI32	F	Hematologic disease	Progressive	3	3.8 (L) 4.7 (R)	11.92	Cochlear C512 (R + L)
CI33	F	Unknown	Progressive	Unknown	6.2 (R) 9.4 (L)	12.08	Cochlear Freedom (R) C512 (L)
CI34	F	Genetic	Progressive	5	15.5 (R)	16.92	Cochlear C512 (R)
CI35	M	Genetic	Progressive	3	6.1 (R) 10.1 (L)	12.33	Cochlear Freedom (R) C512 (L)
CI36	M	Genetic	Progressive	3.5	24.6 (L)	26	Advanced Bionics Naida (L)
CI37	M	Genetic	Progressive	3	19 (L)	20.8	Cochlear Freedom (L)
CI38	F	Genetic-Connexin	Progressive	0.58	13 (R)	23.8	Advanced Bionics Naida (R)
CI39	F	Unknown	Progressive	3	14.8 (L)	22.8	Cochlear Nucleus (L)
CI40	F	Genetic	Progressive	2	16 (R) 19.1 (L)	25.4	Advanced Bionics Neptune (R) Harmony (L)

L = left ear, R = right ear, CMV = Cytomegalovirus.

**Table 2:** Mean age and scores in the linguistic and cognitive tests for the early-implanted, late-implanted, and progressive CI users.

		Age	Raven (%)	TMT (Seconds)	Digit Range (Number)	Semantic Fluency (Number)	Phonemic Fluency (Number)	Receptive Vocabulary (%)
**Progressive**	Mean	16.25	83	22.84	3.84	6.85	13.08	67.10
	SD	6.5	12.77	10.51	1.21	2.92	3.98	18.51
	*n*	13	11	13	13	9	9	9
**Early-Implanted**	Mean	17.75	82.63	22.71	4.35	6.48	12.92	71.14
	SD	5.75	9.32	7	1.39	3.26	3.66	14.25
	*n*	16	15	16	14	9	9	9
**Late-Implanted**	Mean	27.17	74.53	23.5	4.45	8.94	17.83	77.02
	SD	3.75	16.3	10.87	1.21	3.32	4.57	19.22
	*n*	11	8	8	11	6	6	5

Raven = Raven’s Standard Progressive Matrix test, TMT = Trail Making Test (part A), SD = standard deviation.

## Appendix A

**Table A1:** Studies from the past two decades that tested speech-in-noise (SIN) recognition in cochlear implant (CI) users with prelingual deafness.

Study	Participants	Purpose	Method of Testing SIN	SIN Results
Bugannim et al., 2019	NH & CI Young-adults	Assess the effect of auditory training on SIN perception	Hebrew Matrix test sentences in speech-shaped noise; adaptive SNR	CI: Mean SRTn of +1.3 ± 0.6 dB, range: −3.7 to +14 dB. NH: Mean SRTn of −8.1 ± 0.5 dB, range: −10.1 to −6.3 dB
Davidson et al., 2019	CI Children	Identify an optimal level & duration of acoustic experience to facilitate language development	Lexical Neighborhood test (LNT) Words in four-talkers noise; fixed SNR of + 8	CI: 63% correct identification
Goldsworthy & Markle 2019	NH, HA, & CI Children	Assess the effect of different types of noise on SIN perception	Words in speech-spectrum noise, 2-talker babble, and instrumental music; adaptive SNR	CI: Mean SRTn of −5.7, −1.2, & −13.3 dB. NH: Mean SRTn of −9.3, −11.4 & −23.8, for the speech-spectrum noise, 2-talker babble, and instrumental music, respectively
Mishra & Boddupally, 2018	NH & CI Children	Assess the effect of working memory training on SIN perception	Digit-triplets in speech shaped noise; adaptive SNR	CI: Mean SRTn of 15.52 dB, range: +9 to +21.01 dB NH: Mean SRTn of −8.81 dB, range: −11.50 to −6.50
Ching et al., 2018	CI Children	Assess factors that influence SIN perception	Words in a closed-set & BKB: open-set sentences test in babble noise; adaptive SNR	CI: Mean SRTn of 4.0–6.9 dB
Choi et al., 2017	Bilateral & bimodal CI Children	Compare performance between bimodal & bilateral	Words in babble noise; fixed SNR of +5	Bilateral CI: 52.7% ± 25.9% correct identification, Bimodal CI: 40.7% ± 28.7% correct identification
Cusumano et al., 2017	Prelingually & postlingually deafened CI adults	Characterize the performance plateau after unilateral cochlear implantation	HINT or AzBio sentence tests; fixed SNR of +10	Prelingual CI range: from 0% to 90% correct identification at 3 months, 1-year and 2-year post implantation testing
Eisenberg et al., 2016	CI Children (CDaCI study)	Investigate associations between speech perception & spoken language	HINT-C sentences in speech shaped noise; fixed SNRs of +5 & +10	CI: 52% of the sample had achieved >50% correct identification in the +10 and +5 SNR conditions at 3-year post activation testing
Friedmann et al., 2015	CI Adolescents	Examine factors affecting outcomes for sequential bilateral CI	HINT sentence test; fixed SNR of + 10	CI: 92.8% correct identification with both CIs
Van Wieringen & Wouters 2015	CI Children	Assess Predictive factors for spoken language, and auditory & speech perception	CVC words in speech-weighted noise; adaptive SNR	CI: SRTn range from −6 dB to +8 dB
Caldwell & Nittrouer, 2013	NH & CI Children	Examine phonological, language, and cognitive skills in CI children	Words in flat spectrum noise; fixed SNRs of −3, 0, & +3	CI: 0% correct identification at −3 & 0 SNRs, and 13% at +3 SNR. NH: 22%, 27% and 50% correct identification at −3, 0, and +3 SNRs
Kim et al., 2013	CI Children	Assess speech perception in children with a long interval between two implants	Monosyllabic words in speech noise; fixed SNR of +10	CI: Approximately 82% & 85% correct identification for the 1st CI and both Cis respectively
Zeitler et al., 2012	CI Adolescents	Assess the efficacy of implantation in prelingually deafened adolescents	HINT sentences; fixed SNR of +10	No raw % correct data (showing only % change between assessments)
Gifford et al., 2011	NH & CI Children	Assess speech perception with SmartSound strategies	HINT sentences in semi-diffuse restaurant noise; adaptive SNR	CI: Mean SRTn of 14.4 dB and 10.9 dB, depending on the coding strategy. NH: Mean SRTn of 0 dB
Davidson et al., 2011	CI Adolescents	Assess speech perception & correlations to speech production & language tests	BKB sentences in babble noise; fixed SNR of +10	CI Mean: 52.0% ± 26.3% correct identification
Shpak et al., 2009	CI Children, adolescents and young-adults	Assess the benefits of late implantation in prelingually deafened individuals	CID test: sentences in speech-shaped noise; fixed SNR of +10	CI Mean: 34% correct identification two years post implantation
Galvin et al., 2007	CI Children	Evaluate the additional perceptual benefit from sequential bilateral implants	Spondee words discrimination in speech-shaped broadband noise; adaptive SNR	CI: Mean SRTn −12 dB, range: approximately −9 to +1.8 for the 1st CI, and −13 to −4 for both CIs
Wolfe et al., 2007	CI Children	Evaluate speech recognition following sequential implantation	Spondee words in steady state speech-weighted noise; adaptive SNR	CI: Mean SRTn −5.75 dB for the 1st implanted ear, −2.17 dB for the 2nd and −11.75 dB for both CIs
Uziel et al., 2007	CI Children	Assess speech perception, speech intelligibility, receptive language level & academic/occupational status	Meaningful sentences in noise; fixed SNR of +10	CI Mean: 44.5% ± 28% correct identification, range: 0%–94%
Dettman et al., 2004	CI Children	Assess speech perception & bilateral-bimodal benefits for children with significant residual hearing	BKB sentences in multi-talker babble; fixed SNR of +10	CI Mean: 61.71% correct identification

NH = normal hearing, SNR = signal-to-noise ratio, SRTn = the SNR at which 50% of the words in noise are correctly repeated. HINT = Hearing in Noise Test, HINT-C = Hearing in Noise Test for Children, CVC = consonant-vowel-consonant, CID = Central Institute of the Deaf, BKB = Bamford-Kowal-Bench.

## References

[1] O’Donoghue G. (2013). Cochlear implants—Science, serendipity, and success. N. Engl. J. Med..

[2] Perez R., Kishon-Rabin L., Kountakis S.E. (2013). Cochlear Implants–Pediatric. Encyclopedia of Otolaryngology, Head and Neck Surgery.

[3] Bugannim Y., Roth D.A., Zechoval D., Kishon-Rabin L. (2019). Training of Speech Perception in Noise in Pre-Lingual Hearing Impaired Adults with Cochlear Implants Compared with Normal Hearing Adults. Otol. Neurotol..

[4] Caldwell A., Nittrouer S. (2013). Speech perception in noise by children with cochlear implants. J. Speech Lang. Hear. Res..

[5] Ching T.Y., Zhang V.W., Flynn C., Burns L., Button L., Hou S., McGhie K., Van Buynder P. (2018). Factors influencing speech perception in noise for 5-year-old children using hearing aids or cochlear implants. Int. J. Audiol..

[6] Eisenberg L.S., Fisher L.M., Johnson K.C., Ganguly D.H., Grace T., Niparko J.K., Team C.I. (2016). Sentence Recognition in Quiet and Noise by Pediatric Cochlear Implant Users: Relationships to Spoken Language. Otol. Neurotol..

[7] Hick C.B., Tharpe A.M. (2002). Listening effort and fatigue in school-age children with and without hearing loss. J. Speech Lang. Hear. Res..

[8] Mishra S.K., Boddupally S.P. (2018). Auditory Cognitive Training for Pediatric Cochlear Implant Recipients. Ear Hear..

[9] Wilson B.S., Dorman M.F. (2008). Cochlear implants: A remarkable past and a brilliant future. Hear. Res..

[10] Gifford R.H., Shallop J.K., Peterson A.M. (2008). Speech recognition materials and ceiling effects: Considerations for cochlear implant programs. Audiol. Neurotol..

[11] Fu Q.J., Galvin J.J. (2008). Maximizing cochlear implant patients’ performance with advanced speech training procedures. Hear. Res..

[12] Kronenberger W.G., Colson B.G., Henning S.C., Pisoni D.B. (2014). Executive functioning and speech-language skills following long-term use of cochlear implants. J. Deaf Stud. Deaf Educ..

[13] Anderson S., Kraus N. (2010). Sensory-cognitive interaction in the neural encoding of speech in noise: A review. J. Am. Acad. Audiol..

[14] Best V., Gallun F.J., Carlile S., Shinn-Cunningham B.G. (2007). Binaural interference and auditory grouping. J. Acoust. Soc. Am..

[15] Rubinstein J.T. (2004). How cochlear implants encode speech. Curr. Opin. Otolaryngol. Head Neck Surg..

[16] Lorenzi C., Gilbert G., Carn H., Garnier S., Moore B.C. (2006). Speech perception problems of the hearing impaired reflect inability to use temporal fine structure. Proc. Natl. Acad. Sci. USA.

[17] Drennan W.R., Rubinstein J.T. (2008). Music perception in cochlear implant users and its relationship with psychophysical capabilities. J. Rehabil. Res. Dev..

[18] Rönnberg J., Lunner T., Zekveld A., Sörqvist P., Danielsson H., Lyxell B., Dahlström O., Signoret C., Stenfelt S., Pichora-Fuller M.K. (2013). The Ease of Language Understanding (ELU) model: Theoretical, empirical, and clinical advances. Front. Syst. Neurosci..

[19] Stenfelt S., Rönnberg J. (2009). The signal-cognition interface: Interactions between degraded auditory signals and cognitive processes. Scand. J. Psychol..

[20] Boothroyd A. (1997). Auditory development of the hearing child. Scand. Audiol. Suppl..

[21] Spehar B., Goebel S., Tye-Murray N. (2015). Effects of Context Type on Lipreading and Listening Performance and Implications for Sentence Processing. J. Speech Lang. Hear. Res..

[22] Kishon-Rabin L., Boothroyd A., Ravid D., Baron A. (2018). The Role of Hearing for Speech and Language Acquisition and Processing. Handbook of Communication Disorders: Theoretical, Empirical, and Applied Linguistic Perspectivess.

[23] AuBuchon A.M., Pisoni D.B., Kronenberger W.G. (2019). Evaluating Pediatric Cochlear Implant Users’ Encoding, Storage, and Retrieval Strategies in Verbal Working Memory. J. Speech Lang. Hear. Res..

[24] Cejas I., Mitchell C.M., Hoffman M., Quittner A.L. (2018). CDaCI Investigative Team. Comparisons of IQ in Children with and without Cochlear Implants: Longitudinal Findings and Associations with Language. Ear Hear..

[25] Geers A.E., Hayes H. (2011). Reading, writing, and phonological processing skills of adolescents with 10 or more years of cochlear implant experience. Ear Hear..

[26] Johnson C., Goswami U. (2010). Phonological awareness, vocabulary, and reading in deaf children with cochlear implants. J. Speech Lang. Hear. Res..

[27] Chandramouli S.H., Kronenberger W.G., Pisoni D.B. (2019). Verbal Learning and Memory in Early-Implanted, Prelingually Deaf Adolescent and Adult Cochlear Implant Users. J. Speech Lang. Hear. Res..

[28] Niparko J.K., Tobey E.A., Thal D.J., Eisenberg L.S., Wang N.Y., Quittner A.L., Fink N.E., Team C.I. (2010). Spoken language development in children following cochlear implantation. JAMA.

[29] Nittrouer S., Caldwell-Tarr A., Lowenstein J.H. (2013). Working memory in children with cochlear implants: Problems are in storage, not processing. Int. J. Pediatr. Otorhinolaryngol..

[30] Pisoni D.B., Kronenberger W.G., Roman A.S., Geers A.E. (2011). Measures of digit span and verbal rehearsal speed in deaf children after more than 10 years of cochlear implantation. Ear Hear..

[31] AuBuchon A.M., Pisoni D.B., Kronenberger W.G. (2015). Short-Term and Working Memory Impairments in Early-Implanted, Long-Term Cochlear Implant Users Are Independent of Audibility and Speech Production. Ear Hear..

[32] Davidson L.S., Geers A.E., Blamey P.J., Tobey E.A., Brenner C.A. (2011). Factors contributing to speech perception scores in long-term pediatric cochlear implant users. Ear Hear..

[33] Geers A., Brenner C., Davidson L. (2003). Factors associated with development of speech perception skills in children implanted by age five. Ear Hear..

[34] De Boer J., Thornton A.R. (2008). Neural correlates of perceptual learning in the auditory brainstem: Efferent activity predicts and reflects improvement at a speech-in-noise discrimination task. J. Neurosci..

[35] Song J.H., Skoe E., Banai K., Kraus N. (2011). Perception of speech in noise: Neural correlates. J. Cogn. Neurosci..

[36] Akeroyd M.A. (2008). Are individual differences in speech reception related to individual differences in cognitive ability? A survey of twenty experimental studies with normal and hearing-impaired adults. Int. J. Audiol..

[37] Davidson L.S., Geers A.E., Uchanski R.M., Firszt J.B. (2019). Effects of Early Acoustic Hearing on Speech Perception and Language for Pediatric Cochlear Implant Recipients. J. Speech Lang. Hear. Res..

[38] Geers A.E. (2004). Speech, language, and reading skills after early cochlear implantation. Arch. Otolaryngol. Head Neck Surg..

[39] Lunner T., Rudner M., Rönnberg J. (2009). Cognition and hearing aids. Scand. J. Psychol..

[40] Moberly A.C., Bates C., Harris M.S., Pisoni D.B. (2016). The Enigma of Poor Performance by Adults with Cochlear Implants. Otol. Neurotol..

[41] Rudner M., Foo C., Sundewall-Thorén E., Lunner T., Rönnberg J. (2008). Phonological mismatch and explicit cognitive processing in a sample of 102 hearing-aid users. Int. J. Audiol..

[42] Svirsky M.A., Teoh S.W., Neuburger H. (2004). Development of language and speech perception in congenitally, profoundly deaf children as a function of age at cochlear implantation. Audiol. Neurootol..

[43] Manrique M., Cervera-Paz F.J., Huarte A., Molina M. (2004). Prospective long-term auditory results of cochlear implantation in prelinguistically deafened children: The importance of early implantation. Acta Otolaryngol. Suppl..

[44] McConkey Robbins A., Koch D.B., Osberger M.J., Zimmerman-Phillips S., Kishon-Rabin L. (2004). Effect of age at cochlear implantation on auditory skill development in infants and toddlers. Arch. Otolaryngol. Head Neck Surg..

[45] Kral A., Kronenberger W.G., Pisoni D.B., O’Donoghue G.M. (2016). Neurocognitive factors in sensory restoration of early deafness: A connectome model. Lancet Neurol..

[46] Kral A., Dorman M.F., Wilson B.S. (2019). Neuronal Development of Hearing and Language: Cochlear Implants and Critical Periods. Annu. Rev. Neurosci..

[47] Kral A., Sharma A. (2012). Developmental neuroplasticity after cochlear implantation. Trends Neurosci..

[48] Kraaijenga V.J.C., Ramakers G.G.J., Smulders Y.E., van Zon A., Free R.H., Frijns J.H.M., Huinck W.J., Stokroos R.J., Grolman W. (2019). No Difference in Behavioral and Self-Reported Outcomes for Simultaneous and Sequential Bilateral Cochlear Implantation: Evidence From a Multicenter Randomized Controlled Trial. Front. Neurosci..

[49] Hoppe U., Hocke T., Digeser F. (2018). Bimodal benefit for cochlear implant listeners with different grades of hearing loss in the opposite ear. Acta Otolaryngol..

[50] Hua H., Johansson B., Magnusson L., Lyxell B., Ellis R.J. (2017). Speech Recognition and Cognitive Skills in Bimodal Cochlear Implant Users. J. Speech Lang. Hear. Res..

[51] O’Neill E.R., Kreft H.A., Oxenham A.J. (2019). Cognitive factors contribute to speech perception in cochlear-implant users and age-matched normal-hearing listeners under vocoded conditions. J. Acoust. Soc. Am..

[52] Van Wieringen A., Wouters J. (2015). What can we expect of normally-developing children implanted at a young age with respect to their auditory, linguistic and cognitive skills?. Hear. Res..

[53] Choi J.E., Moon I.J., Kim E.Y., Park H.S., Kim B.K., Chung W.H., Cho Y.S., Brown C.J., Hong S.H. (2017). Sound Localization and Speech Perception in Noise of Pediatric Cochlear Implant Recipients: Bimodal Fitting Versus Bilateral Cochlear Implants. Ear Hear..

[54] Friedmann D.R., Green J., Fang Y., Ensor K., Roland J.T., Waltzman S.B. (2015). Sequential bilateral cochlear implantation in the adolescent population. Laryngoscope.

[55] Wolfe J., Baker S., Caraway T., Kasulis H., Mears A., Smith J., Swim L., Wood M. (2007). 1-year postactivation results for sequentially implanted bilateral cochlear implant users. Otol. Neurotol..

[56] Goldsworthy R.L., Markle K.L. (2019). Pediatric Hearing Loss and Speech Recognition in Quiet and in Different Types of Background Noise. J. Speech Lang. Hear. Res..

[57] Cusumano C., Friedmann D.R., Fang Y., Wang B., Roland J.T., Waltzman S.B. (2017). Performance Plateau in Prelingually and Postlingually Deafened Adult Cochlear Implant Recipients. Otol. Neurotol..

[58] Kim J.S., Kim L.S., Jeong S.W. (2013). Functional benefits of sequential bilateral cochlear implantation in children with long inter-stage interval between two implants. Int. J. Pediatr. Otorhinolaryngol..

[59] Zeitler D.M., Anwar A., Green J.E., Babb J.S., Friedmann D.R., Roland J.T., Waltzman S.B. (2012). Cochlear implantation in prelingually deafened adolescents. Arch. Pediatr. Adolesc. Med..

[60] Gifford R.H., Olund A.P., Dejong M. (2011). Improving speech perception in noise for children with cochlear implants. J. Am. Acad. Audiol..

[61] Shpak T., Koren L., Tzach N., Most T., Luntz M. (2009). Perception of speech by prelingual pre-adolescent and adolescent cochlear implant users. Int. J. Audiol..

[62] Galvin K.L., Mok M., Dowell R.C. (2007). Perceptual benefit and functional outcomes for children using sequential bilateral cochlear implants. Ear Hear..

[63] Uziel A.S., Sillon M., Vieu A., Artieres F., Piron J.P., Daures J.P., Mondain M. (2007). Ten-year follow-up of a consecutive series of children with multichannel cochlear implants. Otol. Neurotol..

[64] Dettman S.J., D’Costa W.A., Dowell R.C., Winton E.J., Hill K.L., Williams S.S. (2004). Cochlear implants for children with significant residual hearing. Arch. Otolaryngol. Head Neck Surg..

[65] Ching T.Y., van Wanrooy E., Dillon H., Carter L. (2011). Spatial release from masking in normal-hearing children and children who use hearing aids. J. Acoust. Soc. Am..

[66] Kollmeier B., Warzybok A., Hochmuth S., Zokoll M.A., Uslar V., Brand T., Wagener K.C. (2015). The multilingual matrix test: Principles, applications, and comparison across languages: A review. Int. J. Audiol..

[67] Hey M., Hocke T., Mauger S., Müller-Deile J. (2016). A clinical assessment of cochlear implant recipient performance: Implications for individualized map settings in specific environments. Eur. Arch. Otorhinolaryngol..

[68] Zaltz Y., Goldsworthy R.L., Kishon-Rabin L., Eisenberg L.S. (2018). Voice Discrimination by Adults with Cochlear Implants: The Benefits of Early Implantation for Vocal-Tract Length Perception. J. Assoc. Res. Otolaryngol..

[69] Brand T., Kollmeier B. (2002). Efficient adaptive procedures for threshold and concurrent slope estimates for psychophysics and speech intelligibility tests. J. Acoust. Soc. Am..

[70] Boothroyd A. (1968). Statistical theory of the speech discrimination score. J. Acoust. Soc. Am..

[71] Kishon-Rabin L., Patael S., Menahemi M., Amir N. (2004). Are the perceptual effects of spectral smearing influenced by speaker gender?. J. Basic Clin. Physiol. Pharmacol..

[72] Kavé G. (2005). Phonemic fluency, semantic fluency, and difference scores: Normative data for adult Hebrew speakers. J. Clin. Exp. Neuropsychol..

[73] Raven J.C., Court J.H. (1998). Raven Manual, Section 1 Standard Progressive Matrices.

[74] Wechsler D. (1991). Wechsler Intelligence Scale for Children-III.

[75] Tombaugh T.N. (2004). Trail Making Test A and B: Normative data stratified by age and education. Arch. Clin. Neuropsychol..

[76] Khan S., Edwards L., Langdon D. (2005). The cognition and behaviour of children with cochlear implants, children with hearing aids and their hearing peers: A comparison. Audiol. Neurootol..

[77] Kos M.I., Deriaz M., Guyot J.P., Pelizzone M. (2009). What can be expected from a late cochlear implantation?. Int. J. Pediatr. Otorhinolaryngol..

[78] Santarelli R., De Filippi R., Genovese E., Arslan E. (2008). Cochlear implantation outcome in prelingually deafened young adults. A speech perception study. Audiol. Neurootol..

[79] Zeitler D.M., Kessler M.A., Terushkin V., Roland T.J., Svirsky M.A., Lalwani A.K., Waltzman S.B. (2008). Speech perception benefits of sequential bilateral cochlear implantation in children and adults: A retrospective analysis. Otol. Neurotol..

[80] DeThorne L.S., Schaefer B.A. (2004). A guide to child nonverbal IQ measures. Am. J. Speech Lang. Pathol..

